# Economics of carbon dioxide capture and utilization—a supply and demand perspective

**DOI:** 10.1007/s11356-016-6810-2

**Published:** 2016-05-18

**Authors:** Henriette Naims

**Affiliations:** IASS—Institute for Advanced Sustainability Studies e.V., Berliner Strasse 130, 14467 Potsdam, Germany

**Keywords:** Carbon capture and utilization, Supply and demand scenarios, Commodity CO_2_, Costs of CO_2_ capture, Circular economy

## Abstract

Lately, the technical research on carbon dioxide capture and utilization (CCU) has achieved important breakthroughs. While single CO_2_-based innovations are entering the markets, the possible economic effects of a large-scale CO_2_ utilization still remain unclear to policy makers and the public. Hence, this paper reviews the literature on CCU and provides insights on the motivations and potential of making use of recovered CO_2_ emissions as a commodity in the industrial production of materials and fuels. By analyzing data on current global CO_2_ supply from industrial sources, best practice benchmark capture costs and the demand potential of CO_2_ utilization and storage scenarios with comparative statics, conclusions can be drawn on the role of different CO_2_ sources. For near-term scenarios the demand for the commodity CO_2_ can be covered from industrial processes, that emit CO_2_ at a high purity and low benchmark capture cost of approximately 33 €/t. In the long-term, with synthetic fuel production and large-scale CO_2_ utilization, CO_2_ is likely to be available from a variety of processes at benchmark costs of approx. 65 €/t. Even if fossil-fired power generation is phased out, the CO_2_ emissions of current industrial processes would suffice for ambitious CCU demand scenarios. At current economic conditions, the business case for CO_2_ utilization is technology specific and depends on whether efficiency gains or substitution of volatile priced raw materials can be achieved. Overall, it is argued that CCU should be advanced complementary to mitigation technologies and can unfold its potential in creating local circular economy solutions.

## Motivations for using CO_2_

In the context of the global climate change debate, the motivation behind the research on CO_2_ utilization seems obvious: If there are possibilities to make use of the industrial CO_2_ emissions that are a major cause of global warming they should be harvested. If recovered CO_2_ emissions can be used as feedstock for industrial production processes the existing resource base could be broadened. Especially in the context of circular economy thinking as promoted by the World Economic Forum (2014) making use of waste emissions offers a promising new perspective. However, the largest part of worldwide industrial emissions is still unregulated and unpriced. Currently, only approx. 6 Gt of the estimated annual 37 Gt of global anthropogenic CO_2_ emissions are regulated by some form of carbon pricing instrument (Le Quéré et al. 2014; World Bank 2014). Meanwhile, in the few existing schemes the emission allowance price is rather low, for example around 8 €/t in the EU Emission Trading Scheme (EEX 2015) and approx. 13 US$/t in the California Cap and Trade Program (California Carbon Dashboard 2015). Consequently, the economic incentives to tackle the CO_2_ problem are largely insufficient. The development of technically, environmentally, and economically viable ways of utilizing CO_2_ as a feedstock for industrial production can imply a complementary route to existing mitigation strategies such as the deployment of renewable energy and other green technologies.

As almost all materials that surround us in our everyday lives are carbon-based, the option to use recovered CO_2_ to substitute fossil carbon sources remains an attractive possibility that could be worth further investigation. In the last years, several public funding programs, e.g., by the US Department of Energy or the German Federal Ministry of Education and Research have encouraged research in this field and already the first technological breakthroughs and advances to a demonstration scale can be observed (Federal Ministry of Education and Research 2014; US DOE n.d.). Even though the first CO_2_-based products are just entering global markets in the near future their number and scale is expected to grow (Aresta et al. 2013). Consequently, based on a literature review, the potential supply and demand of the commodity CO_2_ is presented in this paper to discuss the fundamentals of the commodity CO_2_ from an economic perspective of comparative statics.

Since the utilized CO_2_ in most cases is reemitted at a later point in time a simple aggregation of the used volumes of CO_2_ is not an indicator of ecologic performance (von der Assen et al. 2013). Instead, a detailed environmental analysis is necessary to calculate the real carbon footprint of a certain CCU technology compared to a conventional technology (von der Assen et al. 2015). Indeed, the same principle applies to the business case of CO_2_ utilization. In some cases, using comparatively cheap CO_2_ as a feedstock and replacing more costly and volatile priced fossil-based raw materials can lead to a cost reduction which sets the business case for CCU. However, for those production processes that use CO_2_ still inefficiently or are not competitive to conventional fossil-based production, there is no business case until further research and development or political incentives prove otherwise. While CO_2_ can generally be used in many processes, this paper focuses on potential commodity CO_2_ from industrial capture and does not include biological fixation and conversion via the cultivation of crops or algae for example for making biofuels.

## Supply side: potential sources and cost of CO_2_

The potential sources of waste CO_2_ emissions are numerous. Industrial plants emit CO_2_ in different quantities and at diverse qualities. Several capture technologies can be applied, for example adsorption, absorption, cryogenic separation, or membranes (de Coninck and Benson 2014). The costs of capturing CO_2_ at a certain source depend on the technological efforts that must be undertaken to collect the CO_2_ in the required quality from the industrial exhaust gas. Thus, the costs are largely influenced by the concentration of CO_2_ in the exhaust gas. Moreover, the CO_2_ needs to be purified and any toxic or hazardous chemicals removed (Aresta and Dibenedetto 2010). Furthermore, a larger plant size can lower the investment and operating costs per captured tonne of CO_2_ through economies of scale (Faulstich et al. 2009; Möllersten et al. 2003). Consequently, despite technical feasibility, not all emitting sources represent economically viable options at current conditions.

Table 1 summarizes current data on respective global emission volumes, concentrations, estimated capture rates, and benchmark capture costs per type of source for the largest point sources of CO_2_ based on a literature review. The presented data are based on several selected sources with heterogeneous technological and financial assumptions, calculation methods, and reference years. Therefore, the collected data do not allow for a detailed cost comparison or technological discussion. Nevertheless, it is useful to provide general insights on potential large-scale supply of CO_2_ as a commodity. The presented emission volumes represent recent global direct CO_2_ emissions. The capturable emissions are calculated by applying the estimated lower-bound capture rate as a benchmark to the global emission volume per type of source. The benchmark costs represent minimum cost of CO_2_ captured per tonne that are possible to achieve with a certain benchmark technology, a so-called best practice process. The purity of the recovered CO_2_ depends on the respective process but can be assumed between 95 and 99.9 %. The detailed underlying assumptions are further explained in the following and in Table 2.

**Table 1:** Potential sources of waste CO_2_ (most recent available estimates)

CO_2_ emitting source	Global emissions^a^ (Mt CO_2_/year)	CO_2_ content^a^ (vol%)	Estimated capture rate^b^ (%)	Capturable emissions (Mt CO_2_/year)	Benchmark capture cost^b^(€ _2014_/t CO_2_) [rank]	Groups of emitters
Coal to power	9031^c^	12–15	85	7676	34 [6]	Fossil-based power generation
Natural gas to power	2288^c^	3–10^d^	85	1944	63 [9]	Fossil-based power generation
Cement production	2000	14–33	85	1700	68 [10]	Industry large emitters
Iron and steel production	1000	15	50	500	40 [7]	Industry large emitters
Refineries^e^	850	3–13	40	340	99 [12]	Industry large emitters
Petroleum to power	765^c^	3–8	Not available	Not available	Not available	Fossil-based power generation
Ethylene production	260	12	90	234	63 [8]	Industry large emitters
Ammonia production	150	100	85	128	33 [5]	Industry high purity
Bioenergy^f^	73^d^	3–8^d^	90	66	26 [2]	High purity/power generation
Hydrogen production^f^	54^g^	70–90^h^	85	46	30 [4]	Industry high purity
Natural gas production	50	5–70	85	43	30 [3]	Industry high purity
Waste combustion	60^i^	20	Not available	Not available	Not available	Industry large emitters
Fermentation of biomass^f^	18^d^	100^d^	100	18	10 [1]	Industry high purity
Aluminum production	8	<1^j^	85	7	75 [11]	Industry large emitters

^a^Data from Wilcox (2012) if not indicated otherwise

^b^See Table 2 for literature reference, assumptions, and calculation methods

^c^Data from IEA (2014) based on the largest point sources suitable for capture and not including the emissions of the large amount of emissions that are caused by small decentral point sources in the mobility and residential sector

^d^Data from Metz et al. (2005)

^e^Refineries could include ammonia and hydrogen production. A separate listing is nevertheless interesting to differentiate these two high purity from general refinery CO_2_ streams. The capturable emission data based on the estimated capture rates should ensure that emissions are not included twice

^f^Undisclosed technological assumptions for emissions volumes and CO_2_ content, if not indicated otherwise. For technological assumptions for cost data see Table 2. For bioenergy and fermentation, emission estimates are only for North America and Brazil

^g^Data from Mueller-Langer et al. (2007)

^h^Data for hydrogen from steam methane reformer from Kurokawa et al. (2011)

^i^Data from Bogner et al. (2007)

^j^Data from Jilvero et al. (2014), Jordal et al. (2014)

**Table 2:** Overview of capture cost assumptions of CO_2_ point sources

Literature reference	CO_2_ emitting source	Capture cost (€/t CO_2_) and benchmark case	Reference year	Region and currency	Technology assumptions	Economic assumptions
**Finkenrath (** 2011 **)** *Cost and performance of carbon dioxide capture from power generation*, IEA Working Paper.	**Coal to power**	• Average 34–46 € (43–58 $)/t CO_2_ • *Benchmark case:* pre-combustion integrated gasification combined cycle (IGCC) technology at a 20 % net efficiency decrease • ***CEPCI adjusted 2014 benchmark cost: 34 €*** ^a^	Aligned data for 2010 (original studies from 2006 to 2010)	Summarized OECD data (especially from US and EU), in $	• Average normalized performance data from several studies • Data for generic, new-build, early commercial power plants (no pilots or retrofits) • Most advantageous **capture rates between 85 and 100 %** • Undisclosed CO_2_ compression but for transport at supercritical level (>74 bar) • **CO**_**2**_ **purity above 99.9 %**	• **Costs of CO**_**2**_ **avoided** • Average normalized cost data from several studies • Including levelised cost of electricity (LCOE) • Including capital costs as overnight costs without interest costs • Discount rate of 10 % • Not including emission price • not including R&D costs
	**Natural gas to power**	• Average 64 € (80 $)/t CO_2_ • B*enchmark case:* post-combustion capture from natural gas combined cycle at a 15 % net efficiency decrease • ***CEPCI adjusted 2014 benchmark cost: 63 €*** ^a^				
**US EIA (** 2014 **)** *Assumptions to the Annual Energy Outlook 2014,* Independent Statistics and Analysis.	**Cement production**	• **66 €** (82 $)/t CO_2_	2014 estimates	US six regions, in $	• Undisclosed technological assumptions of data • Background: CO_2_ for EOR/EGR from industrial sources, • The CO_2_ is compressed for pipeline transport, thus 90-120 bar and a **CO**_**2**_ **purity of ≥95 % can be assumed** (Metz et al. 2005) • source of cost estimates: US EIA Office of Energy Analysis • ***undisclosed capture rate, thus 85-100 % are assumed*** ^a^	• **Costs of capture and compression** • Including regional transport costs (for EOR/EGR purposes) • not including interregional transport costs • unclarity about inclusion of capital costs
	**Ammonia production**	• **32 €** (40 $)/t CO_2_				
	**Hydrogen production**	• **30–37 €** (37–47 $)/t CO_2_				
	**Natural gas production**	• **30 €** (37 $)/t CO_2_				
**Kuramochi et al. (** 2011 **)** *Techno-economic assessment and comparison of CO*_*2*_ *capture technologies for industrial processes: preliminary results for the iron and steel sector,* Energy Procedia.	**Iron and steel production**	• 40–50 €/t CO_2_ with a large sensitivity to energy prices • *Benchmark case:* vacuum pressure swing adsorption (VPSA) from top gas recycling blast furnace (TGRBF) with a net efficiency decrease due to an increased power consumption • ***CEPCI adjusted 2014 benchmark cost: 40€*** ^a^	Aligned data for 2008	Undisclosed region but reference to several international studies, in €	• Normalized performance data from several studies • Normalized technical parameters: pressure, emission factors and plant scale • C**apture rate of 50 %** • CO_2_ compression to 110 bar • The CO_2_ is compressed for transport, thus a **CO**_**2**_ **purity of ≥95 % can be assumed** (Metz et al. 2005)	• C**osts of CO**_**2**_ **avoided** • Average normalized cost data from several studies: fuel, electricity and capital cost • Including capital costs as total capital requirement (TCR) • Capture costs are very sensitive to energy prices
**van Straelen et al. (** 2010 **)** *CO*_*2*_ *capture for refineries, a practical approach,* International Journal of Greenhouse Gas Control.	**Oil refineries**	• 90–120 €/t CO_2_ for a number of large flue gas sources in oil refining • Above 160 €/t CO_2_ from a large number of scattered, small concentration sources • *Benchmark case:* capture with an amine-based solvent from a combined stack • ***CEPCI adjusted 2014 benchmark cost: 99 €*** ^a^	2007	Western Europe, in €	• Techno-economic case study: post-combustion capture at one large-scale complex oil refinery • **Capture rate of 40–50 %** • Excluding emissions from hydrogen production (approx. 5–20 %) and a large number of small concentration sources (approx. 50 %) • The CO_2_ is compressed for transport and storage, thus 90-120 bar and a **CO**_**2**_ **purity of ≥95 % can be assumed** (Metz et al. 2005)	• C**osts of CO**_**2**_ **avoided** • Including costs of capture and compression • Including capital costs based on a Shell-internal cost estimation tool • Discount rate of 7 % • Excluding transport cost
**Weikl and Schmidt (** 2010 **)** *Carbon capture in cracking furnaces,* AIChe 2010 Spring Meeting and 6th Global Congress on Process Safety.	**Ethylene production**	• 60 € (85 $)/t CO_2_ with a net efficiency decrease due to additional power and steam consumption • ***CEPCI adjusted 2014 benchmark cost: 63 €*** ^a^	Not specified, assumed 2010	No specified region, in $ and €	• Techno-economic case study: post-combustion and oxyfuel capture at one ethylene plant • **Capture rate of 90–99 %** • 20–30 % higher overall emissions • CO_2_ compression to 100 bar • **CO**_**2**_ **purity: >98 % with post-combustion; 85 % with oxyfuel purposes**	• **Costs of capture and compression** • Including fuel, steam and electricity costs • Including capital costs but with high uncertainty • Excluding transport costs • Annuity method with a discount rate of 8 %
**Möllersten et al. (** 2003 **)** *Potential market niches for biomass energy with CO*_*2*_ *capture and storage—Opportunities for energy supply with negative CO*_*2*_ *emissions,* Biomass and Bioenergy.	**Bioenergy**	• 18–42 € (23–53 $)/t CO_2_ • *Benchmark case:* pre-combustion CO_2_ capture in a pulp mill with black liquor integrated gasification combined cycle (BLGCC) technology • ***CEPCI adjusted 2014 benchmark cost: 26 €*** ^a^	Not specified, assumed 2003	No specified region, in $	• Techno-economic case study: post-combustion and IGCC capture at a pulp mill and an ethanol plant • C**apture rate of 90 %** • CO_2_ compression to 100 bar • The CO_2_ is compressed for pipeline or tanker transport, thus a **CO**_**2**_ **purity of ≥95 % can be assumed** (Metz et al. 2005)	• **Costs of capture and compression** • Including lost electricity production • Including capital cost • Excluding transport cost
	**Fermentation**	• 7 € (9 $)/t CO_2_ • *Benchmark case*: only CO_2_compression necessary • ***CEPCI adjusted 2014 benchmark cost: 10 €*** ^a^			• Techno-economic case study: capture from sucrose fermentation in a sugar cane-based ethanol production plant • For non-pressurized fermentation vessels except compression no processing of CO_2_ is needed • **Capture rate of 100 %** • CO_2_ compression to 100 bar • The CO_2_ is compressed for pipeline or tanker transport, thus a **CO**_**2**_ **purity of ≥95 % can be assumed** (Metz et al. 2005)	• **Capture cost only correspond to compression cost** • Excluding capital costs
**Jilvero et al. (** 2014 **)** *Techno-economic analysis of carbon capture at an aluminum production plant—Comparison of post-combustion capture using MEA and ammonia,* Energy Procedia.	**Aluminum production**	• 74–97 €/t CO_2_ • *Benchmark case*: post-combustion CO_2_ capture with ammonia as a solvent at a new aluminum plant (with an increased CO_2_ concentration in the flue gas of 10 %) • ***CEPCI adjusted 2014 benchmark cost: 75 €*** ^a^	2013	Norway, in €	• Techno-economic case study: reference aluminum plant in Norway with post-combustion capture by amines and ammonia • C**apture rate of 85 %** • Additionally emitted CO_2_ from capture is not included • CO_2_ compression for transport to 70–100 bar • **CO**_**2**_ **purity of 99.5 %**	• **Costs of capture and compression** • Net present value (NPV) method combined with Aspen process and cost software • Including capital costs with 25 % contingency fund • But excluding retrofitting costs • Discount rate of 7.5 %

^a^Adjusted/estimated by the author, not included in the cited original literature

### Costs of CO_2_ capture

Today, CO_2_ capture is technologically feasible and industrial practice on a small scale around the world. However, due to a lack of incentives, large-scale capture is currently not economically viable. Hence, the costs of capture are essential when considering potential sources and technologies for recovering CO_2_ emissions. Capture costs are generally defined as the costs of CO_2_ separation and compression at a single facility (e.g., an industrial plant), disregarding any costs of transport, storage, or further conversion steps (Metz et al. 2005). They are usually derived from comparing a system with CO_2_ capture to a reference system without capture. In the literature, two main measures for CO_2_ capture costs exist: costs of capture and costs of avoidance of CO_2_. According to the IPCC (Metz et al. 2005), the two measures are clearly defined as follows:

- *Cost of CO*_*2*_ *captured* represents the rather straightforward cost of capturing per amount of captured CO_2_. They can be determined through formula (1):

1 $$ \begin{array}{l}\mathrm{cost}\;\mathrm{of}\;{\mathrm{CO}}_{2\;}\;\mathrm{capture}\mathrm{d}\;\left(\frac{{\textsf{C}\hspace{-1.7ex}{=}} }{{\mathrm{tCO}}_2}\right)=\frac{\mathrm{additional}\;\mathrm{cost}\mathrm{s}\;\mathrm{of}\;{\mathrm{CO}}_2\;\mathrm{capture}\;\left({\textsf{C}\hspace{-1.7ex}{=}} \right)}{\mathrm{amount}\;\mathrm{of}\;{\mathrm{CO}}_2\;\mathrm{capture}\mathrm{d}\;\left({\mathrm{tCO}}_2\right)}\\ {}\begin{array}{ccc}\hfill \hfill & \hfill \hfill & \hfill =\frac{\mathrm{cost}{\mathrm{s}}_{\mathrm{capture}\;\mathrm{plant}}\;\left({\textsf{C}\hspace{-1.7ex}{=}} \right)-\mathrm{cost}{\mathrm{s}}_{\mathrm{reference}\;\mathrm{plant}}\;\left({\textsf{C}\hspace{-1.7ex}{=}} \right)}{{\mathrm{CO}}_2\;\mathrm{capture}\mathrm{d}\;\left({\mathrm{tCO}}_2\right)}\hfill \end{array}\end{array} $$

These capture cost can reveal “the viability of a CO_2_ capture system given a market price for CO_2_ (as an industrial commodity)” (Bogner et al. 2007). Consequently, if these costs can be reimbursed, e.g., through CO_2_ utilization options or political incentives such as a carbon tax, then carbon capture could make economic sense. Due to this information value, costs of capture are the preferred measure in this study.

(b) *Cost of CO*_*2*_ *avoided* gauges the effect of the overall emission reduction by calculating the cost of capturing per amount of CO_2_ reduced compared to a reference process. The respective formula is (2):

2 $$ \begin{array}{l}\mathrm{cost}\kern0.28em \mathrm{of}\kern0.28em {\mathrm{CO}}_2\kern0.28em \mathrm{avoided}\kern0.28em \left(\frac{\mathsf{C}\kern-1.7ex =}{{\mathrm{tCO}}_2}\right)=\frac{\mathrm{additional}\kern0.28em \mathrm{cost}\mathrm{s}\kern0.28em \mathrm{of}\kern0.28em {\mathrm{CO}}_2\kern0.28em \mathrm{capture}\kern0.28em \left(\mathsf{C}\kern-1.7ex =\right)}{\mathrm{amount}\kern0.28em \mathrm{of}\kern0.28em {\mathrm{CO}}_2\kern0.28em \mathrm{reduction}\kern0.28em \left({\mathrm{tCO}}_2\right)}\\ {}\begin{array}{ccc}\kern2em & \kern2em & \kern1em =\frac{\mathrm{cost}{\mathrm{s}}_{\mathrm{capture}\kern0.28em \mathrm{plant}}\kern0.28em \left(\mathsf{C}\kern-1.7ex =\right)-\mathrm{cost}{\mathrm{s}}_{\mathrm{reference}\kern0.28em \mathrm{plant}}\kern0.28em \left(\mathsf{C}\kern-1.7ex =\right)}{{\mathrm{CO}}_2\kern0.28em \mathrm{emitte}{\mathrm{d}}_{\mathrm{reference}\kern0.28em \mathrm{plant}}\kern0.28em \left({\mathrm{tCO}}_2\right)-{\mathrm{CO}}_2\kern0.28em \mathrm{emitte}{\mathrm{d}}_{\mathrm{capture}\kern0.28em \mathrm{plant}}\kern0.28em \left({\mathrm{tCO}}_2\right)}\kern1em \end{array}\end{array} $$

The cost of CO_2_ avoided considers actual emission reductions and thus contains more ecologically relevant information. As the process of capturing usually requires additional energy and decreases the plant’s efficiency, the capturing often produces additional CO_2_ emissions. Therefore, the amount of CO_2_ reduced or avoided compared to a reference system will largely be smaller than the amount of CO_2_ captured. Consequently, the cost of CO_2_ avoided will generally be higher than the cost of capture (Metz et al. 2005). In an optimal capturing case, where additional energy and emissions can be avoided, both measures will be equal.^1^ The IPCC recommends the cost of avoidance especially for complete carbon capture and storage (CCS) systems and less for capture only analysis (Metz et al. 2005). For the market perspective of this paper, the value of the commodity CO_2_ is however more important. Moreover, it is recommended, that any utilization technology should be accompanied by an LCA that measures the full ecologic impact of all production steps.

Unfortunately, the two measures are often commingled in the literature, so that a clear differentiation is not always possible. The preferred cost estimate of this study is the cost of CO_2_ captured. Deviating cost measures were included where necessary. As described above, the cost of CO_2_ avoided are potentially higher than the capture costs since the reduced emissions in the denominator are smaller than the captured emissions for most capture systems. The difference depends on the additional amounts of CO_2_ emissions caused by the capturing efforts and how they are accounted for. This cost difference is expected to increase more or less proportionally with the efforts that are necessary for capture at the respective sources. Thus, for sources with higher capture costs, the difference in costs of capture and avoidance should be larger. Moreover, even if a measure is clearly and consistently selected in an assessment, varying assumptions and system boundaries limit the possibility of comparing cost data across studies (Metz et al. 2005). Instead, a comparison would only make sense, if the calculation methods and underlying assumptions were fully transparent and either consistent or could be aligned. To improve the measurement quality and comparability of capture costs—within a single study as well as across studies—the reference systems with and without capture should ideally be based on the same assumptions. Firstly, general conditions such as reference year, region, and type of data (real or hypothetical) should be identical at best. Secondly, the technical and economical parameters such as production process, plant size, fuel type, energy cost, and efficiency standards should be comparable. Especially the measuring of capital costs which can strongly influence the capture costs should be consistent.

In a cross-technology comparison, meeting these standards is often not possible. Especially, when emerging technologies and future scenarios are evaluated, reliable and consistent data can be scarce. Instead, a more heuristic approach must be adopted and the best available data analyzed. Thus, this paper summarizes the recent techno-economic literature on carbon capture in order to establish a large-scale picture of CO_2_ supply in the near-term. A secondary database for the largest industrial CO_2_ emitting sources is established in Table 2 that gathers the most recent and reliable cost data available. The presented measures and assumptions are heterogeneous and the data should be considered as estimates and benchmark values for best practice processes. To maximize cost data quality, recent peer-reviewed as well as broader government studies were preferably selected. Other studies were included to fill data gaps. The origin and relative assumptions of the capture cost studies are detailed in Table 2 as far as they were disclosed. Since capture from coal- and natural gas-fired power plants has been discussed the most extensively in the literature, the summarized average costs from the IEA study seem a reliable data source. Moreover, the capture costs for the higher concentrated sources of ammonia, hydrogen, and natural gas as well as cement production derived from the annually updated assumptions of US Energy Information Agency (EIA) seem a reliable data source for the purpose of analysis even though the data regionally cover only the USA. For the other potential sources of CO_2_, less research has been performed and average cost data are not available. Thus, recent peer-reviewed techno-economic studies have been included for capture from iron and steel, refineries, bioenergy, fermentation, and aluminum production. For capture from ethylene production a non-governmental, non-peer reviewed data source was included.

The original data have been adjusted slightly to allow for a common depiction in € per tonne on a 2014 basis. Firstly, cost data in US$ have been converted to € based on Oanda exchange rates^2^ from the end of the respective year of data reference. Then, data that were older than 2014 were adjusted with the annual Chemical Engineering Plant Cost Index (CEPCI). The CEPCI is a composite index that reflects the development of equipment, construction labor, buildings and engineering and supervision costs over time since the 1960s (Chem. Eng. 2008; Chem. Eng. 2015a; Jenkins 2015; Mignard 2014). Thus, it helps to correct for changing economic conditions for chemical plants over time while slightly adjusting the original cost data from older reference years.

### Evaluation of potential CO_2_ supply

Based on the presented data in Tables 1 and 2, a benchmark CO_2_ supply base can be established as a merit order and insights on the feasibility of selected utilization scenarios can be derived. The benchmark CO_2_ supply is based on the best practice minimum capture costs. Upper limit or maximum costs are not presented since it is assumed that a variety of processes exist that could lead to even higher capture costs than those presented in Table 2. The supply cost function thus can be described by formula (3) that sorts and aggregates the capturable quantities of the potential sources (*q*_*i*_) according to their benchmark capture costs (*p*_*i*_):

3 $$ p(q)={p}_i\forall q\in \left]{q}_{i- 1};\;{q}_i\right] $$

*For the space* U *as the union of all intervals*

$$ {\displaystyle {\bigcup}_{i=1}^n\left]{q}_{i-1};\ {q}_i\right]} $$

*with*

*i = rank of CO*_*2*_ *emitting source*

*n = number of ranked sources*

*p*_*i*_ = *benchmark capture cost of source ranked in ith position; in* € *per tonne*;

*p*_1_ ≤ *p*_2_ ≤ … ≤ *p*_*n*_

*q*_*i*_ *= aggregated capturable emissions of sources ranked* ≤ *i; in Mt/year*

For the following considerations, the scenarios will be split in a near-term view of up to 10 years and a long-term horizon of more than 10 years. This differentiation is considered useful for technology development since it is a common assumption that new industrial technologies can grow from lab to commercial scale within 10 years. For near-term scenarios, the presented current volumes and costs of CO_2_ capture seem an adequate estimate. For future volumes of CO_2_ emissions, numerous scenarios exist for different policy scenarios and time horizons, most notably those of the IPCC and the IEA. Future capture costs will vary depending on changing overall economic conditions and energy prices. Improved technological efficiency and performance usually decreases costs over time when the technologies are deployed (Finkenrath 2011). Assuming that capture technologies are advanced further in the future, at stable economic conditions current benchmark costs can likely be lowered or at least maintained.

As assigned in Table 1, the CO_2_ point sources can be divided into four major groups of emitters:

I. *High purity sources*

**Fig. 1:**
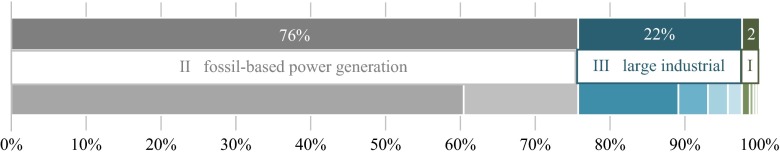
Groups of capturable CO_2_ emissions from large industrial point sources (based on Table 1, 100 % correspond to 12.7 Gt CO_2_)

For certain industrial processes such as ammonia production, the CO_2_ emitted is very pure and capture requires only small additional efforts (IEA 2011 and UNIDO 2011). Therefore, these processes yield relatively cheap CO_2_ as an output. These high-concentration sources represent only approx. 2 % of the 12.7 Gt capturable point source emissions (see Fig. 1). Today, capture of CO_2_ is an established process predominantly in hydrogen, ammonia, and natural gas purification plants as they allow for comparatively cost efficient CO_2_ separation (Wilcox 2012). While raw natural gas can contain CO_2_ in different concentrations depending on the respective source, the processing of the gas to achieve pipeline quality often includes carbon dioxide separation (Baker and Lokhandwala 2008).

II. *Fossil-based power generation*

The largest CO_2_ emitting group—the combustion of coal and gas for power generation—currently is responsible for approx. 76 % of the 12.7 Gt capturable emissions from point sources (see Fig. 1). However, CO_2_ capture at power plants is often connected to significant efficiency losses of approx. 10–30 % of the output energy (de Coninck and Benson 2014; Finkenrath 2011). Consequently, coal and natural gas power plants currently lack business incentives for large-scale capture. If CCS was incentivized by political regulation (e.g. via emission performance standards) power companies could start to implement capture technologies for new plants and possibly retrofits. For power plants, economies of scale can play an important role for lowering capital and operating capture costs per tonne.

III. *Large industrial emitters*

Large industrial CO_2_ emitting processes together currently make up for approx. 22 % of the 12.7 Gt capturable emissions from point sources (see Fig. 1). They include the production of industrial materials such as iron and steel, cement, aluminum as well as refineries. As these processes emit CO_2_ in different quantities and qualities, CO_2_ capture at such plants is also connected to varying efficiency penalties and benchmark costs. Moreover, a large number of other industrial manufacturing plants are potential candidates for CO_2_ capture. Often, they are comparatively smaller than power plants (Bennaceur et al. 2008; Faulstich et al. 2009; Weikl and Schmidt 2010). Thus, economies of scale can be more difficult to achieve. For example, waste incineration so far has barely been analyzed in regard to CO_2_ capture although reusing such CO_2_ would conceptually close resource cycles. The comparatively small size of the incinerators however entails higher capture costs per tonne than those of other CO_2_ sources (Faulstich et al. 2009).

IV. *Natural wells*

It must be noted, that part of the current market is covered by CO_2_ from natural wells instead of recovered CO_2_ emissions (Aresta and Dibenedetto 2010). For example, in the USA, approx. 45 Mt of CO_2_ from natural reservoirs are currently used in enhanced oil or gas recovery (EOR/EGR) (Wilcox 2012). The cost of natural CO_2_ is connected to the oil price (US EIA 2014) and relatively low at 15–20 €/t due to its often rather high purity (Aresta and Dibenedetto 2010). However, assuming that CCU technologies must be measured in regard to their environmental performance, the use of natural CO_2_ carries certain disadvantages. Extracting CO_2_ that is naturally stored underground for the purpose of using it in the production of fuels and materials will result in higher total emissions than when using CO_2_ that is emitted anyway, e.g., by an industrial plant. Thus, it is recommended to replace CO_2_ from natural wells currently in use with recovered CO_2_ to achieve a net emission reduction (Aresta and Dibenedetto 2010; Metz et al. 2005). Since this paper focuses on recovered CO_2_ emissions, CO_2_ from natural sources is consequently not further included in the presented data.

**Fig. 2:**
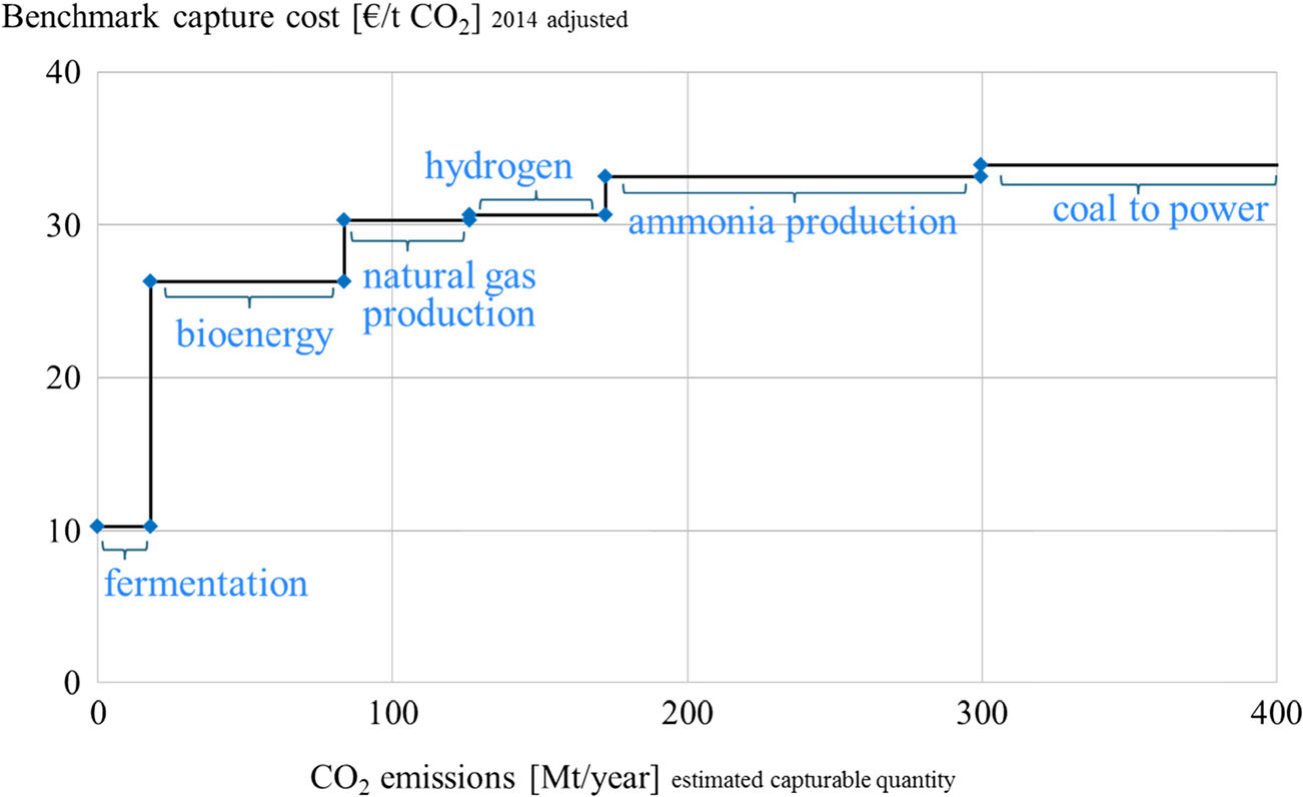
CO_2_ supply curve: high purity and low capture cost sources

**Fig. 3:**
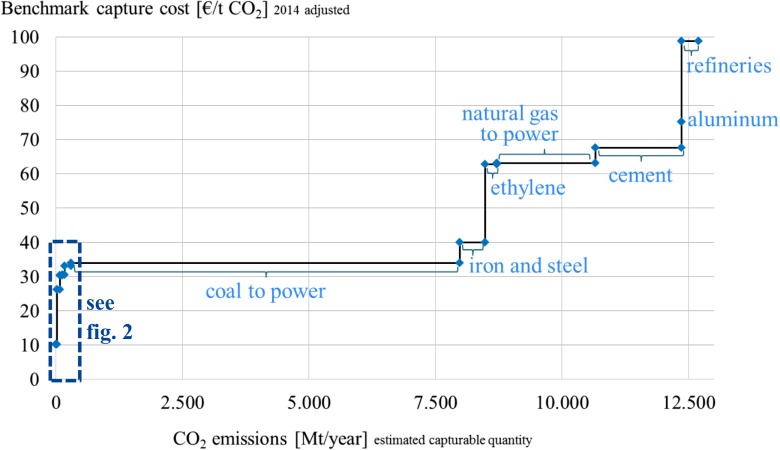
CO_2_ supply curve: fossil power and large industrial sources

Based on the presented emission and cost data a potential supply curve for the commodity CO_2_ is established in Figs. 2 and 3. The low cost sources represent largely the high purity emitters (group I) and are detailed in Fig. 2. These can collectively provide approx. 300 Mt of CO_2_ at a benchmark capture cost of approx. 33 €/t or less. The aggregated capturable CO_2_ supply of 12.7 Gt CO_2_ from all listed point sources is displayed in Fig. 3. Coal power plants can provide large amounts of CO_2_ at relatively low benchmark capture costs of approx. 34 €/t. Nevertheless, even if coal power was phased out in the future, several other industrial processes would cause large amounts of CO_2_ emissions that can be captured at benchmark costs below 100 €/t. Such a supply curve can also be designed for a single plant where different processes emit CO_2_ at various capture costs, as exemplified in van Straelen et al. (2010).

## Demand side: carbon dioxide capture and utilization

The idea of using CO_2_ as feedstock is as old as the chemical industry (Aresta and Dibenedetto 2010), but so far very few applications have been realized. The conversion of CO_2_ with a catalyst evolved in the 1970s, when chemical engineers first succeeded in developing catalysis processes inspired by nature’s CO_2_ conversion cycles (Aresta and Dibenedetto 2010; Aresta et al. 2013). Due to the oil crises at that time, the discovery of alternative feedstock to lower the dependency of fossil resources was economically very attractive. With rising political and public awareness on climate change a large field of research has developed around possibilities to reduce industrial CO_2_ emissions. Accordingly, technological research on CCU technologies slowly but surely has gained momentum in the last decades.

CO_2_ can either be used directly or as feedstock for a variety of products. Overall, approx. 222 Mt of the commodity are used in industrial applications worldwide (see current est. volumes in Table 3). Firstly, direct utilization of liquid or gaseous carbon dioxide usually requires a very high purity especially in the food and beverage industry which currently consumes approx. 11 Mt CO_2_ per year. Furthermore, around 6 Mt CO_2_ are used as process gas in various industrial applications (IHS 2013). The largest direct use of 25 Mt of CO_2_ can be found in EOR/EGR which represent a borderline case, as they combine a utilization and storage function (Global CCS Institute 2014). Largely, they are attributed to CCS rather than CCU since after the extraction of additional fuels through CO_2_, the CO_2_ can potentially be stored permanently in the depleted oil and gas fields. As EOR/EGR is a potential market for recovered CO_2_, it needs to be included when analyzing market volumes of CO_2_ (see Table 3). Secondly, the conversion of CO_2_ to materials still is limited to few applications at a smaller scale, except for urea synthesis which globally currently consumes approx. 130 Mt CO_2_ per year. Indeed, urea and ammonia production are often combined, so that an estimated half of the high purity CO_2_ from ammonia production is used for urea synthesis while the rest is often vented (IEA 2013; Metz et al. 2005). Apart from that, a marginal amount of CO_2_ is used for the production of several specialty chemicals, e.g., of salicylic acid used for making aspirin pills. Commercial plants producing CO_2_-based fuels currently can be found only at demonstration scale of several thousand tonnes, e.g., by the companies Carbon Recycling International (CRI) in Iceland and Audi and Sunfire in Germany (CRI 2016; Strohbach 2013; Sunfire 2014). As R&D on CCU technologies continues and some important breakthroughs have been observed further CO_2_-based products are expected to enter global markets soon as depicted in the near-term (up to 10 years) estimates in Table 3. Thus, the demand for CO_2_ as a commodity might increase in the future.

**Table 3:** Current and near-term markets of CO_2_ utilization (based on Aresta et al. (2013) if not indicated otherwise)

Product/application	Current est. volumes^a^		Near-term est. volumes^b^	
In kt p.a.	CO_2_	Product	CO_2_	Product
*Direct utilization*	*42,400*		*42,400*	
Beverage carbonation^c^	2900	2900	2900^d^	2900^d^
Food packaging^c^	8200	8200	8200^d^	8200^d^
Industrial gas^c^	6300	6300	6300^d^	6300^d^
Oil and gas recovery (EOR/EGR)^e^	25,000	7–23 % of oil reserve, <5 % of gas reserve^f^	25.000^d^	7–23 % of oil reserve, <5 % of gas reserve^f^
*Materials*	*167,515*		*212,400*	
Urea	114,000	155,000	132,000	180,000
Inorganic carbonates	50,000	200,000	70,000	250,000
Formaldehyde	3500	21,000	5000	25,000
PC (polycarbonates)	10	4000	1000	5000
Carbonates	5	200	500	2000
Acrylates	0	2500	1500	3000
Carbamates	0	5300	1000	6000
Formic acid	0	600	900	1000
PUR (polyurethanes)	0	8000	500	10,000
*Fuels*	*12,510*		*20,000*	
Methanol	8000	50,000	10,000	60,000
DME (dimethyl ether)	3000	11,400	>5000	>20,000
TBME (tertiary butyl methyl ether)	1500	30,000	3000	40,000
Algae to biodiesel	10	5	2000	1000
*Total*	*222,425*		*274,800*	

^a^Current data is based on the 2013 estimates from Aresta et al. (2013)

^b^Near-term data is based on the former 2016 estimates from Aresta et al. (2013) and includes CCU technologies that could be implemented within the next 10 years

^c^Data from IHS (2013), worldwide data without Latin America and Asia except Japan

^d^Estimated as constant by the author, not included in the cited original literature

^e^Data from Global CCS Institute (2014)

^f^Estimate from (Metz et al. 2005)

### CO_2_ utilization and emission reductions

CCS aims to store large amounts of CO_2_ underground for long periods of time—approx. 1000 years (Metz et al. 2005). By contrast, when CO_2_ is used directly or as feedstock for materials and fuels it will be reemitted to the atmosphere depending on the durability of the product, ranging from days to several years. As described earlier, the amounts of CO_2_ used thus do not correspond to the amount of CO_2_ avoided. Each CO_2_ utilization process has a different environmental impact which needs to be determined in a life cycle assessment (von der Assen et al. 2015). The crux lies in the efficiency gains connected to the process: If the fossil raw material consumption of a production process can be reduced by the introduction of a CO_2_-based process the environmental balance can be positive. A recent example illustrates how for polyols used for the production of foams up to 3 t of CO_2_ emissions can be avoided per tonne of CO_2_ used compared to a conventional production process (von der Assen and Bardow 2014). Hence, despite the short durability of CO_2_ utilization compared to storage there is an unknown overall mitigation potential that can possibly be significantly larger than the volumes of CO_2_ utilized. However, for a market perspective, the volumes of the commodity CO_2_ that can be captured and used need to be matched. A judgment in regard to mitigation potential is not possible on that basis. While CCS is a recognized emission reduction instrument and commonly accounted for in existing carbon management schemes CO_2_ utilization per se is not accounted as direct emission reduction. If CCU can lead to reductions in fossil raw material use it is possible that CCU indirectly affects emission accounting just as other efficiency measures.

### Evaluation of potential CO_2_ demand

The presented status quo of CO_2_ utilization has demonstrated the limited demand for the commodity CO_2_. However, ongoing worldwide CCU-related research covers a diverse array of utilization options. In the best case “recycling of CO_2_ from anthropogenic sources provides a renewable, inexhaustible carbon source and could allow the continued use of derived carbon fuels in an environmentally friendly, carbon neutral way” (Mikkelsen et al. 2010). Even in direct utilization innovations are possible that go beyond the substitution of fossil feedstock. As currently shown by the CO_2_-based dry cleaning innovation of the US company CO_2_ Nexus large-scale dry cleaning with CO_2_ could potentially lead to economic and environmental benefits by replacing and reducing the consumption of a combination of valuable raw materials such as water, natural gas, and energy (Madsen et al. 2014). At the same time, replacing existing refrigerants with CO_2_ as a standard coolant in automobile air conditioning systems is currently considered at the European Commission (EurActiv.com 2013; Malvicino 2011). Consequently, even when the CO_2_ is directly used it can potentially substitute various substances that are hazardous or have a higher climate impact (Aresta and Dibenedetto 2010).

Overall, future estimates for the potential of CO_2_ utilization in the literature vary, but all range around the same maximum potential. For chemical materials, the estimated large-scale potential is around 200 Mt CO_2_ p.a. (212 Mt est. by Aresta et al. (2013), 200 Mt by Mikkelsen et al. (2010), 180 Mt est. by VCI (2009), and 115 Mt est. by Metz et al. (2005)). By contrast, the estimated large-scale potential for fuel production with CO_2_ is much bigger with approx. 2 Gt CO_2_ p.a. (VCI 2009). Altogether, large-scale CCU can hence potentially require a maximum of 5–6 % of the estimated 37 Gt of anthropogenic CO_2_ emissions (Le Quéré et al. 2014). In order to build a potential demand for the commodity CO_2_, next to the utilization potential the storage potential must also be taken into account. Thus, CCS targets of the IEA will also be included in the scenarios (IEA 2013). Since EOR/EGR is usually considered as storage, it will be attributed to the CCS and not the CCU scenarios in this study. For direct utilization no long-term estimates exist, but due to the limited volumes currently required even significant demand changes are expected to have a minor impact on overall CO_2_ demand. Consequently, the following five demand scenarios were identified:

A. *CCU current*

This scenario represents the current (2013 est.) CO_2_ demand presented in Table 3. The aggregate CO_2_ demand amounts to approx. 200 Mt CO_2_ excluding EOR/EGR.

B. *CCU near-term*

This scenario includes the expected near-term development of CO_2_ utilization over the next 10 years based on the near-term scenario of Table 3. Next to a continued direct utilization of approx. 17 Mt p.a. (excluding EOR/EGR) the production of some CCU-based fuels consumes an estimated 20 Mt of CO_2_ and the aggregated demand for CO_2_ amounts to 250 Mt p.a. Similar demand scenarios of mixed CCU material and fuel activities have also been projected in the range of 250–350 Mt by Quadrelli and Centi (2011) and 300–400 Mt by Aresta and Dibenedetto (2010) for the medium-term.

C. *CCU and CCS near-term*

This scenario combines the projected shorter-term development of 250 Mt CO_2_ used with 40 Mt CO_2_ stored (Carbon Visuals 2014). A combined 290 Mt CO_2_ thus depict a realistic demand for CO_2_ in the near-term of up to 10 years.

D. *CCU fuels long-term*

This scenario illustrates a large-scale potential of CO_2_ utilization including a transformation of the fuels sector to synthetic CO_2_-based fuels. Due to the relatively low current prices of fossil energy, the transition away from fossil fuels is a larger endeavor however that will take much more than 10 years to implement. Implementing this scenario would be connected to severe infrastructure investments in particular in regard to renewable energy and fuel refineries and thus requires strong political efforts. Consequently, this scenario serves rather as an optimistic long-term vision of CCU with 2300 Mt of CO_2_ (250 Mt for materials and direct use and 2050 Mt for fuel production (VCI 2009).

E. *CCU fuels and CCS long-term*

The combination of the 2300 Mt CO_2_ of scenario D and the 2050 target for CCS deployment according to the IEA (2013) of 7870 Mt CO_2_ provides a long-term overall potential demand of combined use and storage of more than 10 Gt CO_2_.

In summary, scenarios A, B, and C represent realistic, near-term scenarios that show a potential demand of 200–300 Mt CO_2_ and represent less than 1 % of anthropogenic emissions while scenarios D and E with 2–10 Gt show an optimistic, long-term potential of materials, fuels, and storage and consume potentially 5–27 % of the current estimated anthropogenic emissions of 37 Gt (Le Quéré et al. 2014).

## Supply and demand scenarios

As a next step, the merit order of supply of CO_2_ from industrial point sources will be matched with the described demand developments for CCU and CCS. For this, the discussed current and potential demand volumes (*d*) for scenarios A to E are combined with the supply of CO_2_ in order to determine the equilibrium best practice benchmark capture prices (*p* ^*^) for each scenario. These are determined by applying formula (4) to the presented data.

4 $$ {p}^{*}(d)=p(d)={p}_i\;for\;d\in \left]{q}_{i-1};\;{q}_i\right] $$

*with*

*d = current and potential demand volumes for scenarios A to E*

*p* ^*^ *= equilibrium best practice benchmark capture prices*

For simplification purposes, it is assumed that the cost of capture correspond to the price of carbon dioxide paid by the consumer which implies that no profits are made on the commodity CO_2_. The presented scenarios are useful to understand the potential allocation of captured emissions in global supply and demand of CO_2_. The analysis of comparative statics is again grouped into short-term and long-term visions.

### Short-term scenarios

**Fig. 4:**
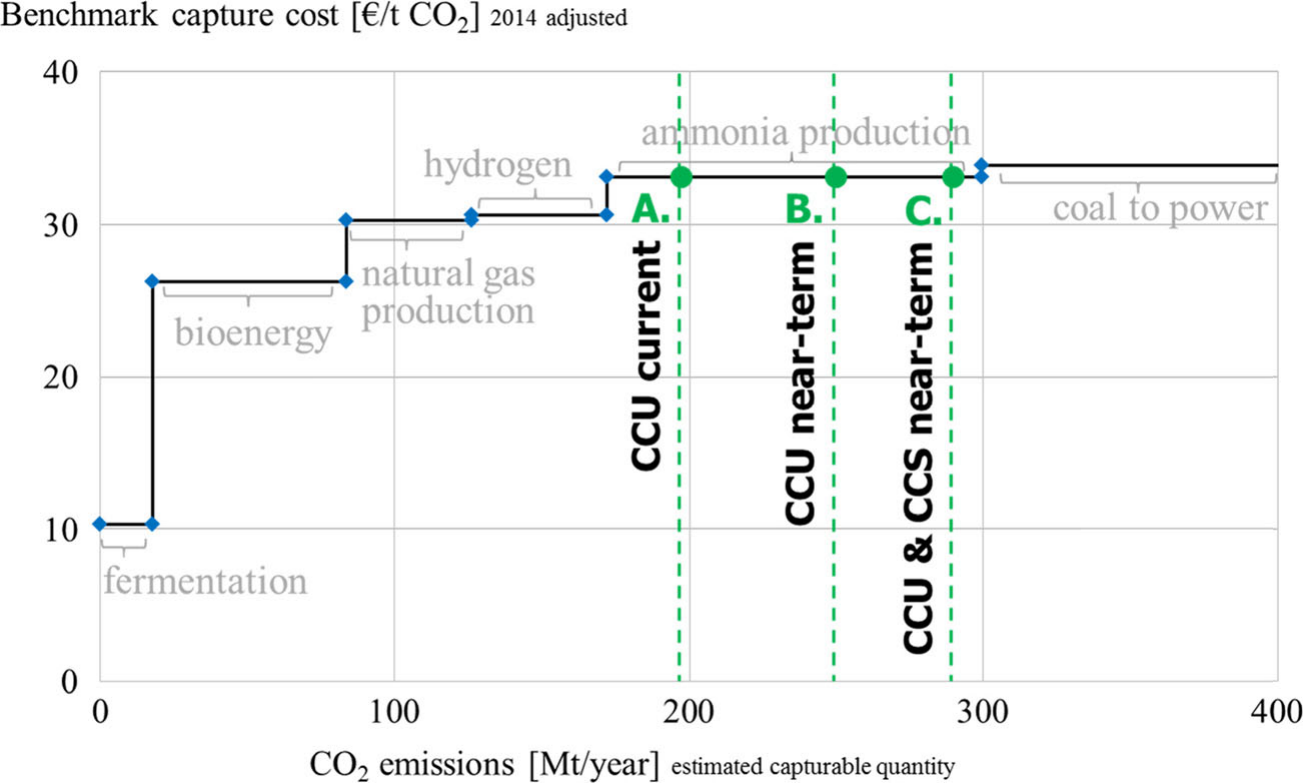
Short-term supply and demand scenarios

The current and near-term potential is depicted in Fig. 4. It becomes evident that there is enough CO_2_ from high purity sources at a comparatively low equilibrium capture cost of approx. 33 €/t to cover the CCU demand in the short-term. From a global perspective, the volumes captured from high purity sources such as fermentation, bioenergy, natural gas production, hydrogen, and ammonia would suffice to cover the current and upcoming CO_2_ demand. Consequently, for the small total volumes required in each specific case the source is usually chosen based on local availability, respective quality, and cost of available CO_2_. A trend towards using a plant’s own or a nearby partner’s waste emissions can be observed at several demonstration plants (Chem. Eng. 2015b; German Embassy Pretoria 2013; Tieman 2013).

### Long-term scenarios

**Fig. 5:**
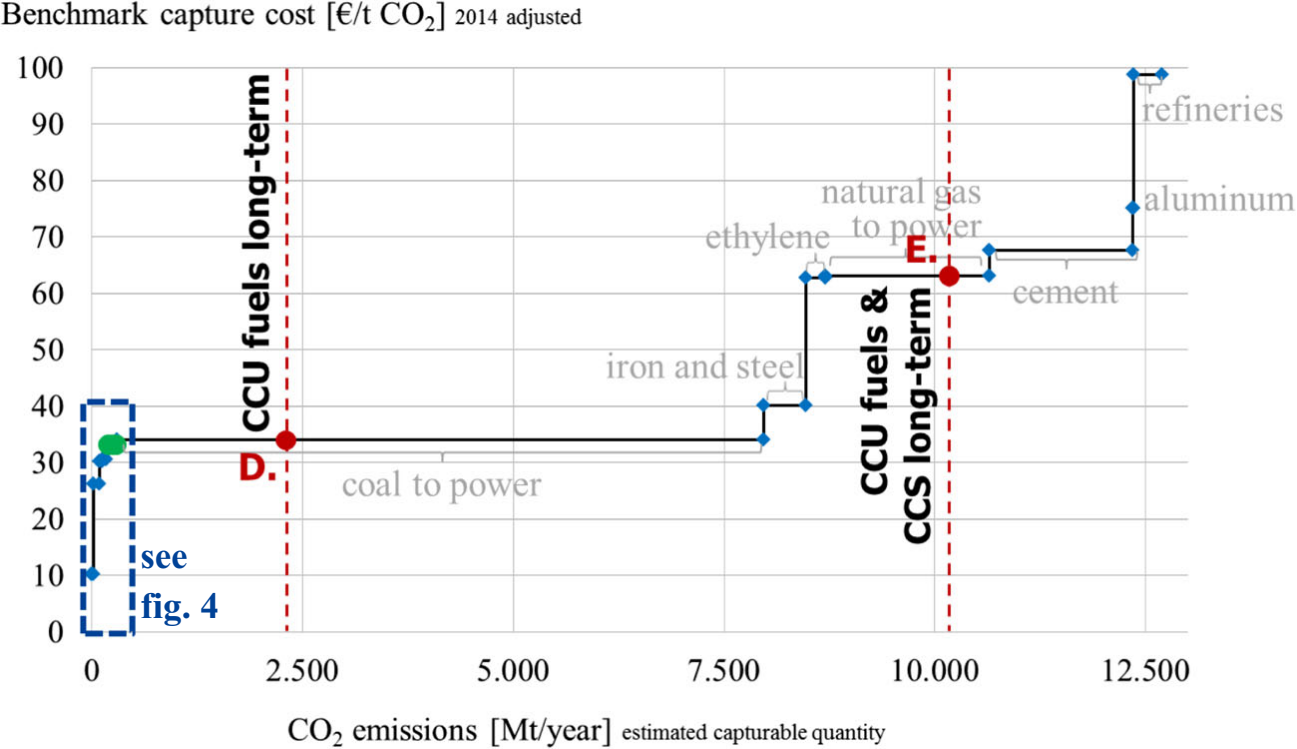
Long-term supply and demand scenarios

Considering the long-term development of CCU and CCS technologies, an optimistic full implementation of described target scenarios D and E is illustrated in Fig. 5. Next to the high purity sources, other industrial plants will become relevant to capture the demanded CO_2_ emissions. Currently, CO_2_ from coal can be captured in large amounts (>7.5 Gt p.a.) at benchmark costs of approx. 33 €/t. Beyond coal and gas, other industrial processes such as iron and steel, ethylene, and cement production can provide CO_2_ at benchmark capture costs of less than 70 €/t. Considering the laid out business as usual supply base the long-term scenario D for CCU including fuels could be achieved with CO_2_ at an equilibrium CO_2_ price of approx. 33 €/t. For a combined long-term target for CCU and CCS, the equilibrium price per tonne based on current conditions would be approx. 63 €/t neglecting possible price changes and inflation.

### Scenarios without fossil-fired power generation

As stated earlier, the future development of CO_2_ emission volumes and prices is unclear due to a variety of political scenarios. The static equilibrium of the current CO_2_ supply with long-term demand scenarios illustrated in Fig. 5 can only provide insights for a business as usual scenario. If however major technological or political changes occur and significantly larger volumes of CO_2_ will continually be mitigated or removed the investments into CCS and potentially also CCU can turn into a dead end and lead to sunk costs in the long run. For future scenarios assuming a larger share of renewable energy, remaining fossil power plants might have to work at lower load factors and efficiencies (Finkenrath 2011). Thus, capture costs at these plants could potentially be higher than the current data suggest. Moreover, for other industrial plants, new technologies can become available in the long run that emit significantly less CO_2_. Then, the relevancy and cost of carbon capture could change—in both directions. For example in the iron and steel production the recently demonstrated Hisarna process can on the one hand reduce approx. 20 % of the conventional CO_2_ emissions. On the other hand, the process allows for a very efficient combination with CO_2_ capture (Pfeifer 2015). Consequently, advancing green technologies across industries will impact available volumes and costs of recovering CO_2_ emissions.

**Fig. 6:**
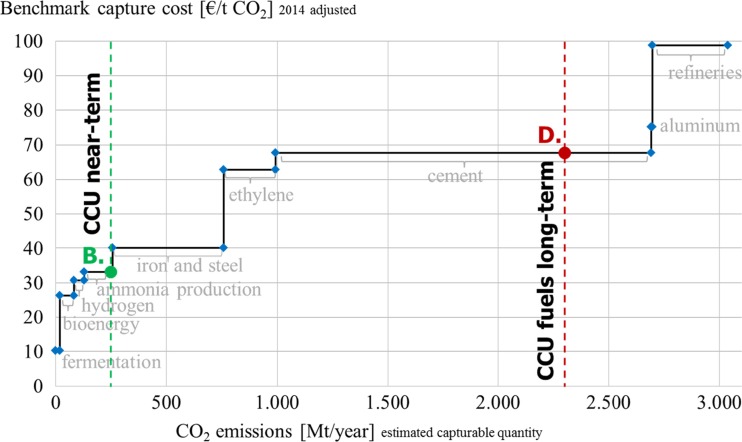
Supply and demand scenario without fossil-fired power generation

Since CCS technologies are largely considered as an instrument to improve the carbon footprint of continued fossil-based power generation (Metz et al. 2005), their implementation becomes less relevant once the energy sector would be based on renewables. To understand the effects on CCU, a modified merit order for CO_2_ supply excluding power generation based on coal and natural gas has been developed in Fig. 6 and matched with a demand scenario excluding CCS. It becomes evident that in the near-term (scenario B) CO_2_ supply for CCU would be provided from industrial high purity sources at an equilibrium price of approx. 33 €/t, in line with scenario B depicted in Fig. 4. Meanwhile the long-term, large-scale potential of CCU including fuels (scenario D) would be served from recovered emissions of various industrial production processes such as iron and steel, ethylene, and cement at equilibrium cost of approx. 68 €/t. This modified supply scenario shows that the future development of CCU technologies is independent from the fossil power industry. By contrast, the amounts of CO_2_ required even for the visionary potential of CCU can possibly be recovered from various industrial sources at estimated costs of less than 70 €/t. CCU technologies thus do not conceptually contradict renewable energy or energy efficiency technologies. Instead, they can be seen as a complementary route.

## Conclusions and perspectives

With carbon capture and utilization, recovered CO_2_ emissions could be turned into a valuable feedstock for the production of consumer goods. CCS in contrast aims to sequester recovered CO_2_ permanently underground. For both technology fields CO_2_ represents a commodity good that is potentially supplied from capture at industrial plants. The comparative statics of CO_2_ demand and supply show that depending on the targeted scenario for CCU and CCS different industrial sources of CO_2_ emissions will play a role: For the near future, smaller scale CCU scenarios, industrial plants with higher CO_2_ concentration and lower benchmark capture costs of approx. 33 €/t will be relevant. Meanwhile, for the long-term large-scale scenarios including the fuel sector and CCS coal-fired power plants or other larger emitters must also be included and CO_2_ captured for benchmark costs of up to 65 €/t will be needed. Even if fossil-fired power generation is phased out, capture at industrial production processes can provide more than enough CO_2_ for large-scale CCU visions. Consequently, reusing recovered industrial CO_2_ emissions can unfold its environmental and economic potential in creating regional or local circular economy solutions. For example, half of the total CO_2_ emissions from waste incineration in Germany would sufficiently supply CO_2_ as a carbon source for the domestic polymer consumption (Bringezu 2014). Moreover, capture of CO_2_ from ambient air remains a potential alternative for recovering CO_2_ emissions. However, technological development is at present still at an early stage and the costs of air capture are highly uncertain and depend on a variety of factors (Lackner et al. 2012). In any case, further research on environmental and economic impacts should accompany the technological advancement of carbon capture and utilization technologies. Harmonizing methods and measures in environmental and techno-economic assessments and establishing best practices would improve the interpretability of the results enormously.

Since the expected near-term status of CO_2_ utilization does not involve large volumes of CO_2_ smaller regional solutions gain importance. When new plants are set up that reuse CO_2_ emissions these can be planned next to a convenient source of CO_2_ at sufficient quality and a competitive cost. Emissions from fossil-fired power plants are not required to meet the potential CO_2_ utilization demand. Even large-scale visions for CCU can therefore not serve as an argument to prolong fossil-fired power generation. When implementing large-scale CO_2_ utilization scenarios involving synthetic fuels based on power-to-liquid or -gas technologies a broader infrastructure especially for renewable energy but also for CO_2_ supply will be needed. Until then, from a mitigation perspective, differentiating recovered CO_2_ by source can even be misleading as in sum it does not play a role to the environment where the reused CO_2_ comes from. Instead, market mechanisms will balance supply and demand. Nevertheless, sustainability aspects always need to be considered when further deploying CCU technologies for example by conducting lifecycle analysis and considering alternative technologies based on renewable energy and raw materials.

Moreover, if energy efficient CCU technologies can be developed, the presented CO_2_ supply benchmark cost range of 10–100 €/t CO_2_ can prove to be a relatively cheap alternative feedstock compared to more expensive or volatile priced chemicals based on fossil raw materials such as crude oil. The search for such technologies that use CO_2_ to replace fossil raw materials and their derivatives consequently remains attractive even in times of relatively low or decreasing energy prices. Currently, in existing emission trading schemes, the CO_2_ is largely underpriced. At current conditions, investments into CCU or CCS hence can only allow for future profits if substitution of expensive raw materials is possible, increased process and production efficiencies can be achieved or government subsidies compensate potential losses. In the future, however, the combined total of approx. 6 Gt of global CO_2_ emissions that are currently regulated by some form of carbon pricing instrument (World Bank 2014) could increase in amount and price. A sufficiently high carbon tax or emission trading price could then incentivize further CCU and CCS activities in certain regions. If the combined costs of capture, transport, and storage of a certain emitting source are lower than the CO_2_ tax or certificate price CCS will have a business case. For example, the relatively old as well as high Norwegian carbon tax, especially for offshore petroleum businesses of up to 69 €/t CO_2_ in 2014 (World Bank 2014) has led to several investments by the affected players into CCS (de Coninck and Benson 2014) and energy efficiency (Bruvoll and Larsen 2004). In contrast, CCU technologies—as efficiency measures generally—are indirectly impacted by the carbon price. Depending on respective energy requirements and prices, certain technologies are profitable even at current conditions. A higher carbon price can be expected to stimulate the deployment of more CCU technologies. Best practice benchmark capture cost can give an indication but do not include potential substitution or efficiency effects connected to CCU processes. An equilibrium price of carbon dioxide for enabling the implementation of CCU from an economic perspective is thus technology specific.

Eventually, while policy makers and businesses must continue to work on mitigating global emissions, pathways for using waste emissions should be pursued complementary. For any desired future, reliable political targets and regulations will be important to permit optimal investment decisions when technologies are implemented and scaled up. Overall, moving ahead, CCU should be considered as a means for improving regional resource security and as enabler of smaller circular economy solutions. Making use of recovered CO_2_ seems to be one option for imitating nature’s no waste philosophy into industrial design and consumption.

## Footnotes

1 This constraint only applies to the capturing system and its reference plant, as well as for CO_2_ storage. If further process steps of CO_2_ utilization are included, the relationship of the two measures can vary in all directions. For example, through raw material substitution or process efficiency improvements the amount of CO_2_ avoided can be larger than the CO_2_ captured. An LCA permits a thorough environmental assessment of utilization options.

2 The Oanda currency converter is available at http://www.oanda.com/currency/converter.
